# Reflections on recruiting healthcare professionals as research participants: Learning from the ONSPres Study

**DOI:** 10.12688/hrbopenres.13499.1

**Published:** 2022-06-27

**Authors:** Sarah Browne, Siobhra Dooley, Aisling Geraghty, Patricia Dominguez Castro, Ciara Reynolds, Carla Perrotta, Lucy Kelly, Kimberley McCallum, Barbara Clyne, Catriona Bradley, Gerard Bury, Sharon Kennelly, Clare Corish, ONSPres Malnutrition Research Study Team

**Affiliations:** 1 School of Public Health, Physiotherapy and Sports Science, University College Dublin, Dublin, Ireland; 2 UCD Institute of Food and Health, University College Dublin, Dublin, Ireland; 3 School of Agriculture and Food Science, University College Dublin, Dublin, Ireland; 4 Health Research Board Centre for Primary Care Research, Department of General Practice, Royal College of Surgeons in Ireland University of Medicine and Health Sciences, Dublin, Ireland; 5 Irish Institute of Pharmacy, Royal College of Surgeons in Ireland University of Medicine and Health Sciences, Dublin, Ireland; 6 School of Medicine, University College Dublin, Dublin, Ireland; 7 National Primary Care Division, Health Service Executive, Mountmellick Primary Care Buildings, Co. Laois, Ireland

**Keywords:** Healthcare professional recruitment, qualitative research, e-learning programme, professional development, malnutrition, oral nutritional supplement prescribing, multi-disciplinary research, primary care

## Abstract

The involvement of healthcare professionals (HCPs) as research participants is essential to generate high quality evidence for enhancing health services and practice.  Research teams face many challenges in recruiting HCPs for research, and barriers and enablers for interdisciplinary research are not well described in the literature.  The Oral Nutritional Supplement Prescribing Malnutrition Research Study (ONSPres Study) examined malnutrition identification, management, and appropriate oral nutritional supplement prescribing in primary care in Ireland.  The ONSPres Study offers a unique view of recruiting HCPs for research because a range of disciplines were sought for participation in a mixed methods study.  The purpose of this open letter is to describe the experiences of recruitment and participation.  Sixteen general practitioners (GPs) were recruited to participate in one-to-one interviews, eighty health and social care professionals working in community care (including nurses, pharmacists, dietitians, physiotherapists, speech and language therapists, and occupational therapists) were recruited to take part in 12 focus groups, and 31 GPs and trainee GPs were recruited to participate in an education programme developed by the study team.   Strategies required to gain access and reach HCPs differed between disciplines.   Professional networks enhanced access to HCPs working in practice and recruitment was slower and more tailored when those networks were less available to the team.  An interest in malnutrition, to assist in research, to advance patient care, and the opportunity for learning were incentives for the participating HCPs.  Limitations in the diversity of the sample arose, with a bias towards female participants and GPs motivated by an interest in the topic.  It is recommended that study teams collaborate early with relevant HCP disciplines so they can contribute to recruitment planning at project concept and design stages.  To enhance and incentivise HCP participation in research, dedicated time and acknowledgement of participation as continuous professional development is proposed.

## Disclaimer

The views expressed in this article are those of the authors. Publication in HRB Open Research does not imply endorsement by the Health Research Board (HRB).

## Introduction

Research in the healthcare sector can successfully determine the best strategies for optimising healthcare services to patients ^1^. It also enables healthcare professionals (HCPs) align health provision with accurate and up-to-date evidence ^2^. HCPs are key stakeholders in health service planning, delivery, and evaluation, and the recruitment of HCPs as research participants is essential to generate high quality and rounded research evidence. 

Barriers to recruitment of HCPs as participants in research include staff time pressures and study burden ^3– 7^. Perceived increase in clinical workload and administration associated with research are considered burdens for HCPs ^3, 8^. Another factor that may inhibit participation is perceived lack of research experience, skill, or knowledge ^5, 9^. To a lesser extent, lack of interest may also be a barrier ^3, 10^ or the perception that in a clinical role, HCPs’ input into research is not necessary or is not valued ^5, 7, 8^.

A key incentive consistently reported by HCPs for being involved in or conducting research is the potential for beneficial patient outcomes ^3, 4, 8, 9^. Enhancing clinical knowledge and skills, and clinical reasoning is also important to HCPs ^3, 4^, as is the potential for career enhancement and recognition ^5, 8^.

Published literature on recruiting HCPs as participants in research has been documented for medical teams and general practitioners (GPs) ^7, 11– 13^. Such insights may be overlooked by other disciplines or may lack applicability to health service research that requires an integrated and interprofessional view. The nature and extent of known or potential barriers and enablers to recruitment may not be fully considered at study design stages or indeed evaluated during implementation. 

The Oral Nutritional Supplement Prescribing Malnutrition Research Study (ONSPres Study) examined malnutrition identification, management, and appropriate oral nutritional supplement prescribing in primary care in Ireland. The ONSPres Study offers a unique view of recruiting HCPs for research because various healthcare disciplines were sought for participation. Findings from interviews with GPs and focus groups with community nurses, staff nurses working in residential care, dietitians, occupational therapists, pharmacists, physiotherapists and speech and language therapists have been published in recent years ^14, 15^, as well as GP evaluation of an e-learning programme ^16^. The purpose of this open letter is to reflect on and share learning about the recruitment processes for a variety of HCP disciplines as part of the ONSPres Study.

## Overview of the ONSPres Study

### Study design

The ONSPres Study was funded by the Health Research Board (HRB) and conducted between 2018 and 2021. Oral nutritional supplement (ONS) use for the treatment of malnutrition was a key element of the study and mixed methods approaches were taken to understand current ONS prescribing practices in Ireland ^14, 17^. The study involved several work packages and data collection methodologies which are described in more detail elsewhere ^15– 23^. The methods relevant to the current paper include qualitative interviews, focus groups and the evaluation of an education programme. GPs, as the primary prescribers of ONS in Ireland, took part in one-to-one interviews that also included a chart-stimulated recall method ^14^. Community dietitians, community nurses, staff nurses working in residential care, occupational therapists, pharmacists, physiotherapists and speech and language therapists were recruited to take part in discipline-specific focus groups ^15^. These qualitative studies were conducted to inform the development of an education programme for HCPs on the identification and management of malnutrition in primary care. A separate cohort of GPs and GP trainees was recruited to participate in a pilot study to evaluate the education (e-learning) programme ^16^. The participating HCP disciplines for each component of the study are detailed in Table 1.

**Table 1.** Healthcare professional disciplines recruited to the ONSPres Study.

Healthcare Professional	N	% Female
**One-to-one Interviews**		
General practitioners	16	75
**Focus Groups (n)**		
Community Dietitian (3)	22	100
Physiotherapist (1)	12	100
Community-based nurses (2)	14	100
Nurses in residential care settings (1)	8	100
Dietitians working with industry (1)	5	100
Pharmacists (2)	9	67
Occupational therapists (1)	6	100
Speech and language therapists (1)	4	100
**E-learning Programme Evaluation**		
General practitioners or trainees	31	60

### Ethics

The study received ethical approval from the University College Dublin Human Research Ethics Committee (Reference number: LS-18-50-Corish) and the Irish College of General Practitioners (ICGP) Research Ethics Committee. Participants were provided with written information about the study and their questions about participation were addressed by the researchers over the phone or by email. Written, informed consent forms were signed and returned to the research team in advance of participation.

### Research team

The research team was led by an academic principal investigator who is a registered dietitian (CC). Two senior post-doctoral researchers (PDC, CR), also registered dietitians, led the recruitment and data collection for interviews and focus groups. A senior post-doctoral researcher (AG), with a background in nutrition research, led the recruitment and data collection for the evaluation of the education programme. The wider ONSPres Study research team included collaborators from the Irish Institute of Pharmacy, HRB (Health Research Board) Centre for Primary Care Research at the RCSI University of Medicine and Health Sciences, Department of Pharmacology and Therapeutics at St James’s Hospital Dublin, School of Public Health, Physiotherapy and Sports Science, Institute of Food and Health, and School of Medicine, all at University College Dublin, and the Health Service Executive (HSE) including representatives from the HSE National Primary Care Division and HSE Medicines Management. Healthcare disciplines represented on the research team included dietetics, general practice medicine, public health medicine, and pharmacy.

## Recruitment of healthcare providers into phases of the ONSPres Study

### GP recruitment for one-to-one interviews

Sixteen interviews were conducted with GPs over a 2-month time frame in 2018 ^14^. Direct referral from within the research team network initiated three GP recruits to the study. The researchers provided soft and hard copies of the recruitment flyer to GPs who participated in interviews, for the purpose of onward sharing. From there, snowball sampling continued the momentum, whereby participating GPs shared the flyer and/or recommended colleagues the research team could contact. Most GPs referred and contacted by the researchers agreed to participate. Time was a barrier for two GPs who agreed to take part when invited, and who ultimately did not participate as the 30-45 minute interview could not be scheduled. Social media recruitment posts did not yield GP participants for the qualitative element of the study; however, was a useful method for GP recruitment for the e-learning programme evaluation discussed below.

### Community pharmacist recruitment for focus groups

As the HCP responsible for dispensing ONS, the views of community pharmacists were important to capture. The research network was also important for recruiting pharmacists; however, snowball sampling did not work as quickly as it did for GPs. In-person, cold calls to eleven pharmacy retailers with recruitment flyers were initiated when other approaches were slow, and four participants for one focus group were recruited. Another focus group was convened from a personal connection to a member of the research team. Organising the interview time and location for community pharmacists was challenging, because participants were coming from multiple locations and businesses. One focus group was cancelled and rescheduled as a minimum number for participation was not achieved on the agreed date. 

### Other healthcare professionals’ recruitment for focus groups

Recruitment for HCPs working within the HSE followed a ‘top down’ approach whereby discipline department managers were contacted by researchers to inform them about the study and discuss the possibility of recruiting within their departments. This required groundwork to identify service managers. Additional recruitment strategies included advertisement via professional representative organisations, special interest groups, and on social media. However, it was direct management contact that was most successful for several reasons. Department managers could encourage staff to participate and allow protected time to do so. The focus groups could be scheduled at a time when staff were together for another reason, for example, at lunchtime after a team meeting. 

Professional networks and within discipline recommendations also played a role in recruiting within the HSE. In the case of community nurses, a contact within the research team yielded the first focus group and, from there, a participating nurse introduced the research team to a colleague in another area. Recruitment of community dietitians was the least challenging in this study as the research was led by registered dietitians working in academia and the HSE, and many service managers were known to the research team. However, dietetic managers also assisted with introductions to service managers from other disciplines and those introductions yielded more positive responses when compared to cold-calling service managers. 

For speech and language therapists, alternative approaches were needed to ensure a minimum number of focus group participants likely to have experience of malnutrition in the community setting. Since many speech and language therapists work in paediatric care, identifying those working specifically in adult care required contact with special interest groups of professional organisations and calling primary care centres to speak directly with department managers or speech and language therapists working onsite.

### GP recruitment to participate in evaluating the e-learning programme

GP representatives on the research team, and general practice colleagues from other universities sent invitations with information about the project and the e-learning programme pilot study. Social media, specifically Twitter, was also used to advertise and accounts linked to Irish GP organisations were tagged within posts to gain traction. Increases in enquiry emails were noted immediately after Twitter ‘drives’. To promote the continuing professional development (CPD) opportunity, advertising content focused on the malnutrition education component for GPs. Recruitment took place in 2020 when the COVID-19 pandemic and social lockdowns were at their height in Ireland. One researcher spent approximately 12 weeks recruiting GPs to take part in the e-learning programme pilot study. Retaining the participants to the end of the evaluation was more challenging and while the majority completed the immediate post-module assessments, just 35% of GPs (n=11) completed the follow-up assessment 6 weeks post-programme. Two to three email reminders were sent to participating GPs to prompt completion. 

### Incentives to participation

GPs taking part in interviews were each given a €120 One4All voucher (accepted in a variety of retail/leisure/hospitality outlets in Ireland) as a token of appreciation and compensation, given participation time was not necessarily protected work time and required time away which may have had an impact on income. One GP did not accept the voucher. GPs participating in the e-learning programme were entered into a draw to win a One4All voucher valued at €150. Other HCPs were given One4All vouchers of lesser value (€20) as tokens of appreciation. Certificates of attendance and participation in the research were provided by the ONSPres study team and these could be used by some HCPs towards CPD credits. Refreshments were provided to HCPs participating in focus groups including lunch (if meeting through lunch time), snacks and beverages. 

An interest in the topic and the learning opportunity were reported incentives for many of the HCP participants. One GP, for example, was motivated to participate as they worked in a socially deprived area and were in regular contact with patients with malnutrition owing to drug addiction, housing shortages, and other social issues. Many participating HCPs expressed an interest in receiving feedback on the study outcomes and evaluation of the e-learning programme. 

Convenience and minimising the time commitment were important considerations in data collection. In the main, research staff travelled to participants to facilitate interviews and focus groups and, in some cases, called to GP homes when this was suggested as most convenient. For GPs taking part in the e-learning programme, the researchers were very clear and exact about the time investment required for completing the programme in advance. Flexibility was also emphasised in that the programme could be completed at a time and location convenient to the GP or GP trainee. 

## Discussion

The experiences of recruiting HCPs in primary care to participate in a study involving interviews, focus groups and a CPD pilot study were summarised in this paper. Different approaches to the recruitment of healthcare disciplines in three strands of the study were utilised. GPs and community pharmacists work independently to the HSE and interdisciplinary collaborations, professional introductions and referrals, and sufficient time to recruit were facilitators. HCPs working within the HSE were facilitated to participate when department or service managers agreed to the research requirements and provided protected time and space for focus groups. Offering educational opportunities and CPD as part of the research was viewed as an incentive to participation; however, with an e-learning programme, there may be challenges with loss to follow-up to evaluate knowledge and skill retention.

Hysong *et al.* ^13^ highlighted difficulties in recruiting clinicians to research and devised a framework for estimating the time and resources needed to recruit HCPs as participants. The framework outlines potential bottlenecks at various steps including gaining entry, obtaining accurate records, reaching participants, assessing willingness to participate, scheduling participants. In 2006 Solberg outlined a framework to guide HCP recruitment which included seven ‘R-factors’: relationship, reputation, requirements, rewards, reciprocity, resolution and respect ^11^. The two approaches are relevant in categorising the experiences of recruiting within the ONSPres study. ‘Gaining entry’ and ‘reaching participants’ were enhanced by reputation of the researchers and professional relationships and hampered without them. Within the ONSPres project team, the principal investigator and a HSE clinical specialist dietitian collaborator have strong track records in community/primary care malnutrition research. Thus, the research team had a sense that dietetic service managers were more willing to support and accommodate research for their own discipline than service managers from other disciplines. Furthermore, the reputation of collaborators from other disciplines and professional introductions were instrumental in gaining access to non-dietetic HCPs. Respect for participants’ time, being clear on the requirements of participation and integrating flexibility to deliver on those factors were important elements of success in finally scheduling participants. As recommended by others ^11, 12, 24^, ongoing adaptations to recruitment strategies were important so that we could respond flexibly to barriers and enablers encountered. If approaching recruitment again, we would place more emphasis on learning and CPD as rewards than financial incentives which has been raised by others recruiting HCPs in primary care ^7, 24^, however considerations should be given to the unique requirements of individual disciplines and their settings. 

While we did not calculate the time and resources dedicated to recruiting, overall recruitment was a time-intensive task which can impact on meeting sample sizes and implementation plans ^7, 12, 13^. Dormandy *et al.* ^25^ reported on facilitators to recruiting primary care practices into research. These included the research topic being perceived as important, relevant and of interest to the participating GPs. Our experience concurs with this and highlights the potential issue of self-selection bias within the sample. GPs and community pharmacists were drawn from large pools of eligible participants, reflecting the large number of GP practices and pharmacy dispensaries across the sampled community health areas. In contrast, the HSE HCP focus groups were comprised of most of the eligible team members working in the community health area. There was a strong bias towards female participation, and this is explained by a dominance within the profession for some disciplines (e.g. nursing and health and social care professions) but not others (GPs, pharmacists) ^26^. In addition, while many of the participating GPs expressed an interest in learning more about malnutrition, this may not reflect the general experience of all GPs. We know from the ONSPres study quantitative analysis that there is a wide variety of ONS prescribing practices, with some GPs not prescribing at all and some showing high levels of prescribing ^23^. Interviews with GPs did not seem to reflect extremes in prescribing and views and experiences on malnutrition identification and management echoed those of other HCPs ^14, 15^. Therefore, we acknowledge the limitations in the convenience sample for qualitative interviews ^27^ and concede that the current reality of recruitment can make it difficult to avoid these limitations. 

### Implications and recommendations for future research

This paper integrates the reflections of the research team on recruiting HCPs at the end of a mixed methods study. Greater insight in terms of enablers and barriers to participation among HCPs would have been possible by involving participants in post-participation surveys or interviews. A barrier to participation among HCPs that is consistently cited in the literature is difficulty in trying to implement unrealistic research approaches into busy clinical practice ^3, 5, 7, 8^. One potential avenue for the future is a systems level change so that participation in health service research is valued and rewarded alongside conducting research for HCPs. This could be supported by employers and professional bodies through incentives, education and CPD recognition for participation and time involved. While there are many productive examples of direct collaborations between academia and health service departments through clinical partnership programmes and awards ^28^ (and for example, the HRB applied partnership awards), researchers on the ground face similar recruitment challenges during projects with sustainability frequently an issue owing to inadequate research supports within clinical sites ^28^. Table 2 summarises key learning from the ONSPres study and recommendations for future research and practice in recruiting HCPs for health service research. 

**Table 2.** Key learning about recruitment during the ONSPres study and recommendations for recruiting healthcare professionals in health service research.

Domain	Key learning and recommendations for future research & practice
**Understanding recruitment** ** of HCP disciplines**	A steering group that includes relevant HCP disciplines can inform study protocol design including access and reach, recruitment processes, time commitments, and incentives to participation.
**Gaining Entry**	Utilising professional networks for introductions enhanced access. Professional and academic reputations assist with credibility, relationships and access.
**Reaching participants**	Recruitment contact included telephone contact, email, visiting pharmacy outlets, and flyer/infographic posts via email and social media. Social media was useful for promoting an educational programme to GPs. A medium that was not utilised was video and given the proliferation of audio-visual to deliver concise and personalised messages online during the COVID-19 pandemic, this approach should be considered by future researchers recruiting HCPs. Forward planning processes for achieving diversity in the sample, as dictated by the research topic, are recommended.
**Assessing willingness to** ** participate**	Interest in the topic, to assist in research, to advance patient care, to improve individual knowledge and skill and to keep up to date with study outcomes are potential motivators for HCPs.
**Incentives / Rewards**	Research teams should explore and document all the potential benefits to participation that can be communicated to HCPs at recruitment. Personalising the benefits of CPD and education within research participation could be emphasised as these incentives were deemed most successful during the ONSPres study. Providing online resources for all participants could be considered to encourage participation in research and offers the advantage of being accessible at any time and location ^29^. The offer of monetary incentives may not be a primary driver of recruitment, however consultation with each target HCP discipline in advance is advised.
**Scheduling participants**	HCPs valued flexibility in terms of scheduling interviews and focus groups at times and locations convenient for them individually or for teams. Clarity regarding the time involved is important to communicate.
**Follow-up and retention**	The e-learning programme had high attrition of GP and GP trainees at 6 weeks. Communications were conducted via email during the COVID-19 pandemic. Phone or video calls may have enabled more rapport building and personal contact that can be helpful in primary care recruitment and retention ^7, 24^. Small group learning, with planned group activities at defined time points, has been widely cited as a successful approach to GP continuing medical education (CME) and CPD and could have been explored in the ONSPres study if time and resources allowed.

HCP – Healthcare Professionals; GP – General Practitioner; CPD – Continuous professional development.

## Conclusion

Recruitment for research can be challenging in all settings and populations. Recruiting HCPs requires early engagement with target disciplines, who ideally work as collaborators within the research team. Professional relationships, strategic introductions, and reputation of the study team are key enablers in recruiting HCPs to research. While some challenges can be anticipated, unforeseen or unplanned barriers will also come into play. Clarity on the benefits of participation to each HCP discipline is important in advance of recruitment. When recruiting HCPs, we recommend offering flexibility where possible and providing a realistic estimate of time involved. Education, CPD recognition and opportunities for learning were incentives to HCP participation in the ONSPres study. Employer and organisational support for research participation among HCPs is recommended as part of CPD.

## Data availability

No data are associated with this article.
