# Supplementary material for: Reflections on recruiting healthcare professionals as research participants: Learning from the ONSPres Study
Source: HRB Open Res. 2022 Jun 27;5:47. [Version 1] doi: 10.12688/hrbopenres.13499.1 (PMC9428496; doi:10.12688/hrbopenres.13499.1)
Supplement: Peer review history

# Peer review: Reviewer response for version 1

**DOI:** 10.21956/hrbopenres.14724.r32660

**Published:** 2022-08-30

**Authors:** Evelina Liljeberg

**Affiliations:** 1 Department of Food Studies, Nutrition and Dietetics, Uppsala University, Uppsala, Sweden; 2 Medical Unit Clinical Nutrition, Women's Health and Allied Health Professionals Team, Karolinska University Hospital, Solna, Sweden

This open letter covers an important topic: reflections of the research team on the recruitment of healthcare professionals (HCPs) as study participants, which seldom receives enough room for discussion in papers describing the main findings of studies. The ONSpres Study is introduced to the reader followed by a description of the different recruitment strategies used for various HCPs. Throughout this open letter, barriers and enablers to the recruitment of HCPs in this project and in general are discussed. Published literature within this field is nicely referred to.

 For a researcher who is very familiar with the papers published from the ONSpres Study, this open letter was very interesting to read and fills an important role when it comes to transferring practical experiences of conducting research to a wider public. I have one minor suggestion for improvement of the article: Table 2 is very informative and plays a main role in this open letter, however, key learnings and recommendations are described together here. There are also points that are neither, for example, the following one under the Domain, Reaching participants: “Recruitment contact included telephone contact, email, visiting pharmacy outlets, and flyer/infographic posts via email and social media”. Can the recommendations in Table 2 be communicated more clearly somehow, e.g. by a column on its own or by some other inventive strategy?

Does the article adequately reference differing views and opinions?

Yes

Are all factual statements correct, and are statements and arguments made adequately supported by citations?

Yes

Is the Open Letter written in accessible language?

Yes

Where applicable, are recommendations and next steps explained clearly for others to follow?

Partly

Is the rationale for the Open Letter provided in sufficient detail?

Yes

Reviewer Expertise:

I am a registered dietitian with a PhD from 2021 who works as a senior lecturer within nutrition and dietetics at Uppsala University and a clinical dietitian within the field of oncology at Karolinska University Hospital, Sweden. My research concerns dietetic ONS prescribing and adherence to this nutrition intervention for disease-related malnutrition. I use quantitative as well as qualitative methods.

I confirm that I have read this submission and believe that I have an appropriate level of expertise to confirm that it is of an acceptable scientific standard.

# Peer review: Reviewer response for version 1

**DOI:** 10.21956/hrbopenres.14724.r32657

**Published:** 2022-08-15

**Authors:** Anne Griffin

**Affiliations:** 1 School of Allied Health, Faculty of Education & Health Sciences, Health Research Institute, University of Limerick, Limerick, Ireland

This open communication will resonate with many research teams involved in the study of healthcare service and delivery. The authors describe the barriers and enablers to recruiting various healthcare professionals from the perspective of their experience of exploring oral nutrition supplement prescribing in primary care. They have adequately contextualised their experience within the available evidence from the published literature. They acknowledge the limitations that exist with recruitment methods that include the membership of the research team itself, a reliance on professional networks, and an understanding of the nature of incentives in return for participation.

 The authors recommend an early collaboration with relevant healthcare professional disciplines commencing, such as engagement from the project conception, planning, and design stage. My one suggestion in relation to recommendations for others is signposting to Campus Engage - resources and frameworks might provide supportive guidelines to research teams about engagement, involvement, and participation in a research project.

Does the article adequately reference differing views and opinions?

Yes

Are all factual statements correct, and are statements and arguments made adequately supported by citations?

Yes

Is the Open Letter written in accessible language?

Yes

Where applicable, are recommendations and next steps explained clearly for others to follow?

Partly

Is the rationale for the Open Letter provided in sufficient detail?

Yes

Reviewer Expertise:

Before joining the University of Limerick, School of Allied Health academic team in 2018, I practised as a registered dietitian with the HSE and private consultancy for 20 years. During this time, I established and provided a community dietetic service and was involved in the development, implementation, and support of programmes to promote health and prevent nutrition-related diseases in a range of clinical & community settings. I continue to work in nutrition health promotion through partnerships I have developed and maintained with both HSE and community development groups. My current research interests include the exploration of nutrition care pathways for older adults, particularly in the transition from emergency departments and faculty development to support interprofessional education.

I confirm that I have read this submission and believe that I have an appropriate level of expertise to confirm that it is of an acceptable scientific standard.
